# Principles of Human Physiology, with Their Chief Applications to Pschycology, Pathology, Therapeutics, Hygiene, and Forensic Medicine

**Published:** 1855-11

**Authors:** 


ART. X.—*Principles of Human Physiology, with their chief applications to Pschycology, Pathology, Therapeutics, Hygiene, and Forensic Medicine.* By WILLIAM B. CARPENTER, M. D., F. R. S., F. G. S., etc.,etc. A new American from the last London Edition. Edited, with additions, by FRANCIS GURNEY SMITH, M. D., Professor of the Institutes of Medicine in the Medical Department of Pennsylvania College, etc. Philadelphia: Blanchard & Lea. 1855.

This is the beginning of a new arrangement of Dr. Carpenter's well known works on physiology. As all our readers are familiar with the merits of the author as a physiological author, it is only necessary that we should announce this new edition and arrangement, that purchasers may govern themselves accordingly.

It will be recollected that Dr. Carpenter has heretofore published numerous editions of two works, separately entitled "Principles of Human Physiology," and "Principles of General and Comparative Physiology." In the course of numerous revisions and additions, the latter work has become so overgrown and cumbersome that it is now to be divided into two distinct treatises on "General" and "Comparative Physiology," respectively. In preparing a new edition of the "Human Physiology," it became evident that it was already too bulky to bear additions, and the constant additions to knowledge in this branch making some additions necessary, the matter has been arranged by transferring from the "Human" to the "General Physiology," all such parts of the former as could properly be incorporated with the latter.

Thus Dr. Carpenter's works will now arrange themselves into three separate treatises on "Human," "General," and "Comparative Physiology," separately.

As this work is the standard one on the subject in this country, and as undoubtedly very many of our readers will supply themselves with it, we have taken the pains to inform them fully of the new arrangement.

It is only necessary to add that the active mind of Dr. Carpenter is evident in the changes and advances to be noticed in the present volume.

